# A systematic review of the protective effects of silymarin/silibinin against doxorubicin-induced cardiotoxicity

**DOI:** 10.1186/s12935-023-02936-4

**Published:** 2023-05-10

**Authors:** Mandeep Singh, Mustafa M. Kadhim, Abduladheem Turki Jalil, Shamam Kareem Oudah, Zafar Aminov, Fahad Alsaikhan, Zanko Hassan Jawhar, Andrés Alexis Ramírez-Coronel, Bagher Farhood

**Affiliations:** 1 Department of Physical Education, University of Jammu, Srinagar, Jammu India; 2 Department of Dentistry, Kut University College, Kut, Wasit 52001 Iraq; 3 Medical Laboratory Techniques Department, Al-Farahidi University, Baghdad, 10022 Iraq; 4 Medical Laboratories Techniques Department, Al-Mustaqbal University College, Babylon, Hilla 51001 Iraq; 5 National University of Science and Technology, Nasiriyah, Dhi Qar Iraq; 6 Department of Public Health and Healthcare Management, Samarkand State Medical University, 18 Amir Temur Street, Samarkand, Uzbekistan; 7 Department of Scientific Affairs, Tashkent State Dental Institute, 103 Makhtumkuli Str., Tashkent, Uzbekistan; 8 College of Pharmacy, Prince Sattam Bin Abdulaziz University, Alkharj, Saudi Arabia; 9 Department of Medical Laboratory Science, College of Health Sciences, Lebanese French University, Erbil, Kurdistan Region Iraq; 10 Clinical Biochemistry Department, College of Health Sciences, Hawler Medical University, Erbil, Kurdistan Region Iraq; 11 Azogues Campus Nursing Career, Health and Behavior Research Group (HBR), Psychometry and Ethology Laboratory, Catholic University of Cuenca, Cuenca, Ecuador; 12 Epidemiology and Biostatistics Research Group, CES University, Medellín, Colombia; 13 Educational Statistics Research Group (GIEE), National University of Education, Cuenca, Ecuador; 14 Department of Medical Physics and Radiology, Faculty of Paramedical Sciences, Kashan University of Medical Sciences, Kashan, Iran

**Keywords:** Cancer, Cardiotoxicity, Doxorubicin, Silymarin, Silibinin, Systematic review

## Abstract

**Purpose:**

Although doxorubicin chemotherapy is commonly applied for treating different malignant tumors, cardiotoxicity induced by this chemotherapeutic agent restricts its clinical use. The use of silymarin/silibinin may mitigate the doxorubicin-induced cardiac adverse effects. For this aim, the potential cardioprotective effects of silymarin/silibinin against the doxorubicin-induced cardiotoxicity were systematically reviewed.

**Methods:**

In this study, we performed a systematic search in accordance with PRISMA guideline for identifying all relevant studies on “the role of silymarin/silibinin against doxorubicin-induced cardiotoxicity” in different electronic databases up to June 2022. Sixty-one articles were obtained and screened based on the predefined inclusion and exclusion criteria. Thirteen eligible papers were finally included in this review.

**Results:**

According to the echocardiographic and electrocardiographic findings, the doxorubicin-treated groups presented a significant reduction in ejection fraction, tissue Doppler peak mitral annulus systolic velocity, and fractional shortening as well as bradycardia, prolongation of QT and QRS interval. However, these echocardiographic abnormalities were obviously improved in the silymarin plus doxorubicin groups. As well, the doxorubicin administration led to induce histopathological and biochemical changes in the cardiac cells/tissue; in contrast, the silymarin/silibinin co-administration could mitigate these induced alterations (for most of the cases).

**Conclusion:**

According to the findings, it was found that the co-administration of silymarin/silibinin alleviates the doxorubicin-induced cardiac adverse effects. Silymarin/silibinin exerts its cardioprotective effects via antioxidant, anti-inflammatory, anti-apoptotic activities, and other mechanisms.

## Introduction

Cancer, known as an uncontrolled growth of cells, is one of the leading causes of death in the world [1–3]. Among current mainstay treatments for cancer include surgery, chemotherapy, and radiotherapy [4–6]. Cancer chemotherapy is the application of drug(s) to cancer patients [7]. Advancements in chemotherapeutic drug discovery have resulted in a remarkable increase in survivorship for cancer patients [8]. However, a number of chemotherapeutic drugs cause adverse effects such as cardiovascular toxicity that may be devastating and life-threatening to cancer patients [9].

Anthracyclines are a class of chemotherapeutic agents that are administered in adult and pediatric patients for treating different cancers [10]. Doxorubicin (also known as Adriamycin) is the most common anthracycline which is widely used to treat different malignant tumors, including acute leukemia, lymphomas, ovarian, testicular, lung, thyroid, breast cancers, and so on [11–15]. Despite its potency, the doxorubicin-associated toxicity on various body organs (particularly the heart) limits its clinical use [16, 17]. Cardiotoxicity is defined as the deterioration of ejection fraction by more than ten percent in asymptomatic cases with a final ejection fraction of less than fifty-five percent or a reduction in ejection fraction of at least five percent in symptomatic cases with a final ejection fraction of less than fifty-five percent [18, 19]. Clinically, doxorubicin-induced cardiotoxicity is characterized by a decrease in the left ventricular ejection fraction, aberrant arrhythmias, and congestive heart failure as well as an increment in the ventricular wall thickness, which can lead to death [10, 20, 21]. This chemotherapeutic drug acutely and chronically causes cardiac adverse effects through induction of oxidative stress, apoptosis and inflammation, mitochondrial dysfunction, inhibition of nucleic acids, and other mechanisms [22–24]. Fortunately, previous studies have reported that the use of combination chemotherapy could alleviate the doxorubicin-induced cardiotoxicity [25, 26]; as the doxorubicin co-administration with other agents having chemoprotective capabilities can enhance the therapeutic efficacy of doxorubicin and mitigate different toxicity to normal cells/tissues at the same time [27, 28].

The use of herbal plants and their derivatives in order to alleviate the chemotherapy-associated toxicity (chemo-protectors) or increase the sensitivity of tumoral cells to chemotherapeutic drugs (chemo-sensitizers) has attracted much attention. Silymarin is a polyphenolic flavonoid mixture extracted from the seeds of *Silybum marianum* [29]. It is noteworthy that the standardized extract of this herbal agent contains various flavonolignans of silybin A, silybin B, silychristin A, silychristin B, isosilybin A, isosilybin B, and silydianin (approximately 65–80%), fatty acids and polyphenolic compounds (approximately 20–35%), and small amounts of flavonoids [30]. Silibinin is also a 50:50 ratio of silybin A and silybin B. It has been confirmed that silibinin is the major bioactive component of silymarin. [31, 32]. Moreover, it was shown that silymarin is one of the best pharmacologically characterized plant extracts because it is non-toxic and without side effects even at relatively high physiological dose values which can be used for treating different diseases [33, 34]. In this regard, silymarin has been used as a natural remedy for nervous system, kidney, prostate, lung, liver diseases, etc. [35, 36]. Among the protective activities of silymarin can point to antifibrotic, immunomodulatory, membrane‐stabilizing [37, 38], antioxidant [39], anti-apoptotic [40], and anti-inflammatory [41] properties. The antitumoral effects of this herbal agent have been assessed in some tumors such as lung, liver, cervical, breast, bladder, skin, and prostate cancers [42–49]. The different mechanisms for the antitumor activities of silymarin have been reported by previous studies [38, 45, 46, 50–54].

To the best of our knowledge, this study is the first systematic review regarding the cardioprotective potentials of silymarin/silibinin, as an adjuvant, against the doxorubicin-induced cardiac adverse effects. In this regard, it was tried to answer the following issues: (a) How does doxorubicin cause cardiotoxicity? (b) What are the underlying mechanisms of cardiac adverse effects induced by this chemotherapeutic agent? (c) What is the role of silymarin/silibinin against the doxorubicin-induced cardiotoxicity? (d) What are the cardioprotective mechanisms of silymarin/silibinin against the doxorubicin-induced cardiac adverse effects?

## Methods

We performed a comprehensive and systematic search in accordance with the Preferred Reporting Items for Systematic Reviews and Meta-Analyses (PRISMA) guideline [55]. In this study, we also used a PICO framework [55] for structuring the review process:

- Participants (**P**): patients/animals with cardiac complications from doxorubicin (for clinical studies/in-vivo experiments) and/or cardiac cells injured by doxorubicin (for in-vitro experiments)
- Intervention (**I**): cardiac cells/patients/animals treated with silymarin/silibinin plus doxorubicin
- Comparison (**C**): cardiac cells/animals/patients treated with doxorubicin
- Outcomes (**O**): there were two main outcomes: (1) the cardiac adverse effects induced by doxorubicin in the cardiac cells/tissue than the control groups and (2) the changes resulted in the cardiac cells/tissue following silymarin/silibinin plus doxorubicin than doxorubicin alone

### Search strategy

A systematic search was carried out for obtaining all relevant scientific papers on “the cardioprotective effects of silymarin/silibinin against the doxorubicin-induced cardiotoxicity” in different electronic databases of Scopus, PubMed, and Web of Science up to June 2022 using the keywords “Silymarin” OR “Milk thistle” OR “Carduus marianus” OR “Silybum” OR “Silybum marianum” OR “Carsil” OR “Silibinin” OR “silybin” OR “Legalon” OR “Marian thistle” OR “Karsil” OR “Blessed milk thistle” OR “Scotch thistle” OR “Mary thistle” OR “variegated thistle” OR “Saint Mary's thistle” OR “Mediterranean milk thistle” AND “Doxorubicin” OR “Adriamycin” AND “Cardiac” OR “Heart” OR “Cardiomyopathy” OR “Cardiopathy” OR “Cardiac Toxicity” OR “Cardiac Toxicities” OR “Cardiopathic” OR “Arrhythmias” OR “Myocardium” OR “Cardiotoxicity” OR “Myocardial” OR “Myocyte" OR “Cardiomyocyte” in the title, abstract or keywords.

### Study selection process

We initially selected all studies based on the study objective (the role of silymarin/silibinin against the doxorubicin-induced cardiotoxicity) in the title and abstract. In the next stage, the full-text papers with (a) English language, (b) adequate findings, (c) no restriction on publication year, and (d) no restriction in publications with in-vivo, in-vitro, or clinical studies were included in the present systematic review. Additionally, we excluded not related papers, book chapters, review papers, case studies, letters to the editors, posters, editorials, and oral presentations from the current study.

### Data extraction

Each eligible paper was independently investigated by two authors (MS and ZHJ). When there was a discrepancy between these two authors, it was resolved by consulting the third author (BF). The following data were then extracted for each eligible study: (a) author name and publication year, (b) models (clinical study, in-vivo experiment or/and in-vitro experiment), (c) dosage, protocol of usage, and administration route of doxorubicin, (d) outcomes obtained from doxorubicin administration on the cardiac cells/tissue, (e) dosage, protocol of usage, and administration route of silymarin/silibinin, and (f) findings obtained from silymarin/silibinin co-administration on the doxorubicin-induced cardiotoxicity.

## Results

### Literature search and screening

**Fig. 1:**
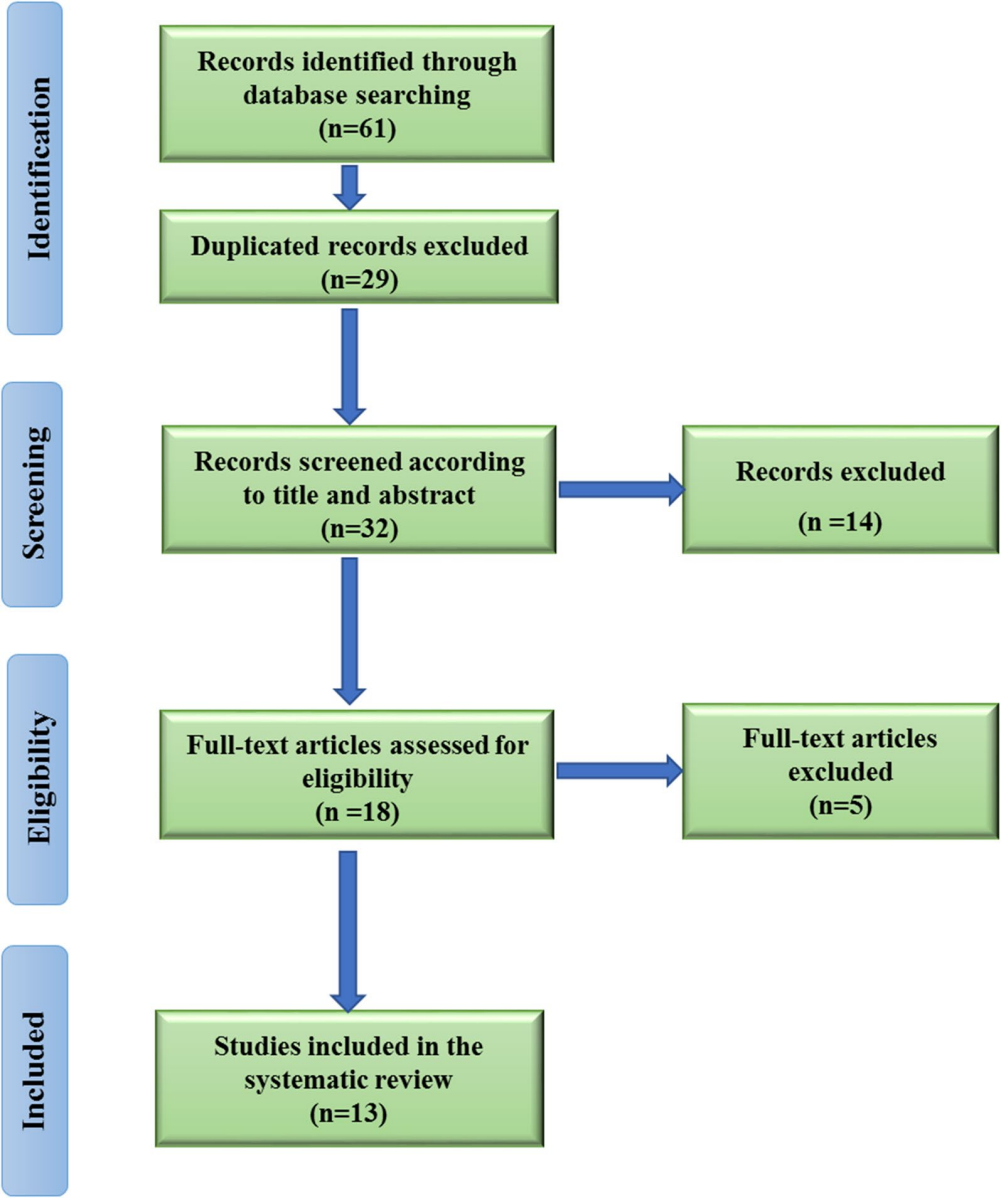
PRISMA flow diagram illustrating the selection process of studies

We obtained sixty-one papers up to June 2022. After removing the duplicate studies (*n* = 29), thirty-two studies were screened in their titles and abstracts. Fourteen studies were then excluded and eighteen remaining studies were qualified for assessment of their full texts. Thirteen studies were finally included in this review. The selection process of the study is also shown in Fig. 1. Furthermore, the findings extracted from thirteen eligible studies are summarized in Table 1.

**Table 1:** The characteristics of included studies

References	Model	DOX dosage and protocol of usage; administration route	Outcomes of DOX on cardiac cells/tissue	*Silymarin/^#^silibinin dosage and protocol of usage; administration route	Silymarin/silibinin co-administration outcomes
Psotova’ et al. [34]	In vitro/rat heart microsomes and mitochondria	77 μmol/L and180 min	–	*9.66 mg/L (IC_50_ for microsomes) and 4.90 mg/L (IC_50_ for mitochondria)	↓LPO (TBARS)
ChlopCíková et al. [67]	In vitro/rat cardiomyocytes	100 μM and 8 h	–	*19.5, 39.0 and 78.0 mg/L and 1 h prior to DOX incubation and ^#^25, 50 and 100 μM and 1 h prior to DOX incubation	↓LDH released activity, ↑ATP formation
El-Shitanyet al. [62]	In vivo/rats	10 mg/kg and single dose on 7th day; *i.p*.	↑plasma CPK and LDH enzyme activities, ↑plasma cholesterol and total lipids concentrations, ↑LPO (MDA), induction of histological damage (sporadic early necrotic fibers, highly eosinophilic cytoplasm, minimal mononuclear cellular infiltration and intravascular, vascular congestion, minimal interstitial edema)	*50 mg/kg/day and starting from 7 days before DOX administration and continued consecutively for 10 days; *i.p*.	↓plasma CPK and LDH enzyme activities, ↓plasma cholesterol and total lipids concentrations, ↓LPO (MDA), alleviation of DOX-induced histological changes
Patel et al. [57]	In vivo/mice	60 mg/kg and single dose on 12th day; *i.p*.	↓body weight, ↑mortality rate, ↑creatine kinase	*16 mg/kg/day and starting from 12 days before DOX administration and continued consecutively for 14 days; oral	↑body weight, ↓mortality rate, ↓creatine kinase
Cecen et al. [63]	In vivo/rats	10 mg/kg and single dose on 5th day; *i.p*.	↑nitric oxide, induction of histological and ultrastructural changes (cytoplasmic vacuole formation and interstitial edema, myofibrillar disorganizations, disintegration and dilatation of sarcoplasmic reticulum, irregular nuclear membrane and vesiculated rough endoplasmic reticulum)	*100 mg/kg/day and starting from 5 days before DOX administration and continued daily until euthanization throughout the project (7 and 21 days after DOX administration); *i.p*.	↓nitric oxide (7 days after DOX administration), alleviation of DOX-induced histological and ultrastructural changes
Rašković et al. [59]	In vivo/rats	1.66 mg/kg/injection and every other day for 12 days; *i.p*.	↓body weight, ↑myocardial excitability, ↑AST, ↑LDH, ↑creatine kinase, mild hyperemia	*60 mg/kg/day and for 12 days; oral	↑body weight, ↓myocardial excitability, ↓AST, ↓creatine kinase
Arozal et al. [58]	In vivo/rats	Cumulative dose of 15 mg/kg and 2.5 mg/kg in six equal injections for two consecutive weeks; *i.p*.	↓survival rate, ↓heart weight, ↑ascites, ↑pericardial, pleural and peritoneal fluids, ↑heart rate, prolongation of QT and QRS interval, ↑CK-MB and LDH, ↑MDA, ↓SOD, induction of histological changes [↑infiltration of inflammatory cells, ↑focal necrosis, ↑fibrosis (%)]	*50 mg/kg/day and starting at the same day of DOX administration and continued for 5 weeks; oral	↑heart weight, shortening of QT and QRS interval, ↓CK-MB and LDH, alleviation of DOX-induced histological changes
Afsar et al. [60]	In vivo/rats	Cumulative dose of 18 mg/kg and 3 mg/k/week for 6 weeks; *i.p*.	↓body weight, ↑creatine kinase, CK-MB, AST and LDH, ↓RBCs, WBCs, Hb%, PCV, MCV, MCH and MCHC values, ↓platelets and neutrophil counts, ↑lymphocyte counts, ↓CAT, peroxidase, SOD, quinone reductase, GSH, γ-GT, glutathione reductase, GST and GPx levels, ↓cardiac tissue protein contents, ↑TBARS, H_2_O_2_ and nitrite contents, induction of histological changes (↑infiltration of inflammatory cells, eosinophilic degeneration, necrotic muscle fibers, hypertrophy of muscle fibers, distorted blood capillaries, interstitial edema, disturbance in cardiac trabeculae, retrogressive lacerations in muscle fibers and vacuolated muscle fibers)	*100 mg/kg/administration and 2 administrations per week for 6 weeks (12 doses/6 weeks); oral	↑body weight, ↓creatine kinase, CK-MB, AST and LDH, ↑RBC, WBC, Hb%, PCV, MCV, MCH and MCHC values, ↑platelets and neutrophil counts, ↓lymphocyte counts, ↑CAT, peroxidase, SOD, quinone reductase, GSH, γ-GT, glutathione reductase, GST and GPx levels, ↑cardiac tissue protein contents, ↓TBARS, H2O2 and nitrite contents, alleviation of DOX-induced histological changes
Attia et al. [64]	In vivo/rats	1.66 mg/kg/injection and every other day for 12 days; *i.p*.	↑VEGF-A, iNOS and caspase-3 expressions, ↑LDH, CPK and AST concentrations, ↓GSH concentration, induction of histological and ultrastructural changes (disturbed general architecture with degenerated cardiac myocytes, areas of severe hemorrhage along with markedly congested blood vessels, degenerated cardiac myocytes with small deeply stained pyknotic nuclei and vacuolated cytoplasm, noticeable hemorrhage between cardiac myocytes bundles, cardiac myocytes having areas of separated and lost myofibrils, cells with increased eosinophilia, ↑collagen fibers with areas of fibrosis between cardiac myocytes, thickening of coronary artery wall, degenerated cardiac myocytes with irregular corrugated thick basement membrane, cardiac myocytes with small shrunken fragmented nucleus, wide spaces in cytoplasm, irregular arrangement and mitochondria shape between remnants of myofibrils, irregular shaped small peripheral nucleus along with peripheral condensation of its chromatin, cardiomyocytes with wide intercellular space containing many fibroblasts and collage fibers)	*60 mg/kg/day and for 12 days; oral	↓VEGF-A, iNOS and caspase-3 expressions, ↓LDH, CPK and AST concentrations, ↑GSH concentration, alleviation of DOX-induced histological changes (normal appearance and arrangement of cardiac myocytes, minimal hemorrhage and congested blood vessels, normal branching and anastomosing cardiac myocytes with central oval vesicular nuclei, normal arranged myofibrils and intercalated discs, few number of cardiac myocytes having areas of myofibrillar loss, ↓collagen fibers in coronary artery wall and between cardiac myocytes bundles, blood vessels with mild condensation of collagen fibers on their walls, cardiac myocytes having rode shaped nuclei along with fine chromatin and regular arranged myofibrils, normal shaped and regular arranged mitochondria with prominent cristae between the myofibrils)
Hagag et al. [61]	Clinical study/acute lymphoblastic leukemia patients	25 mg/m^2^/week and for 6 weeks; *i.v*.	↓ejection fraction, ↓fractional shortening, ↓tissue Doppler peak mitral annulus systolic velocity, ↑serum troponin I level	*420 mg/day for one week after each doxorubicin administration and in the form of Legalon tablet or Hepaticum syrup	↑ejection fraction, ↑fractional shortening, ↑tissue Doppler peak mitral annulus systolic velocity, ↓serum troponin I level
Abdelsalam et al. [65]	In vivo/rats	3 mg/kg and then 2 mg/kg and 2 weeks apart; *i.v*.	↑total cholesterol, triglycerides, LDL-c, TG/HDL, LDL/HDL and C-reactive protein, ↓HDL-c, ↑Nrf2 expression, induction of histological changes (cardiomyocytes with darkly stained nuclei or margination of chromatin, disruption of myocardial architecture, vacuolated or pale sarcoplasm, splitting of cardiac fibers, wide spaces between muscle fibers, loss of cardiomyocyte striations along with deeply stained acidophilic sarcoplasm, severely congested blood vessels and inflammatory exudates)	*600 mg/kg/day and for 4 weeks; oral	↓total cholesterol, triglycerides, LDL-c, TG/HDL, LDL/HDL and C-reactive protein, ↑HDL-c, ↑↑Nrf2 expression, alleviation of DOX-induced histological changes
Akinloye et al. [66]	In vivo/rats	20 mg/kg and single dose on 8th day; *i.p*.	↑MDA, ↑nitric oxide, ↓SOD, CAT, glutathione reductase, GST and GSH levels, ↑TNF-α, ↓IL-10, ↑C-reactive protein, cardiomyocytes with congested coronary blood vessels	*200 mg/kg/day and as pretreatment for 7 days; oral	↓MDA, ↓nitric oxide, ↑SOD, CAT, glutathione reductase, GST and GSH levels, ↓TNF-α, ↑IL-10, ↓C-reactive protein, cardiomyocytes with mild congested blood vessels
Ortona et al. [56]	In vitro/AC16 cell line (derived from adult human ventricular cardiomyocytes)	1 μM and 72 h	↓cell survival rate, ↑cardiomyocyte apoptosis, ↑mitochondrial ROS production, ↑cells (%) with depolarized mitochondria, actin/myosin disorganization, loss of bipolar shape of cardiomyocytes along with compromised orientation of stress fibers	^#^100 μM and 48 h before Dox administration	↑cell survival rate, ↓cardiomyocyte apoptosis, ↓mitochondrial ROS production, ↓cells (%) with depolarized mitochondria, counteraction of DOX-induced cytoskeleton alterations

↑, Increase; ↓, Decease; i.p., Intraperitoneal; DOX, Doxorubicin; LPO, Lipid peroxidation; MDA, Malondialdehyde; ROS, Reactive oxygen species; GPx, Glutathione peroxidase; SOD, Superoxide dismutase; CAT, Catalase; γ-GT, Gamma-glutamyl transferase; GST, Glutathione-S-transferase; IL-10, Interleukin 10; TNF-α, Tumor necrosis factor-alpha; LDH, Lactate dehydrogenase; AST, Aspartate aminotransferase; CPK, Creatine phosphokinase; GSH, Glutathione; CK-MB, Creatine kinase-myocardial band; TBARS, Thiobarbituric acid reactive substances; VEGF-A, Vascular endothelial growth factor A; iNOS, Inducible nitric oxide synthase; LDL-c, Low-density lipoprotein-cholesterol; HDL-c, High-density lipoprotein-cholesterol; TG/HDL, Triglyceride/high-density lipoprotein; Nrf2, Nuclear factor erythroid 2-related factor 2

### The cardioprotective potentials of silymarin/silibinin on the doxorubicin-induced cardiac adverse effects

#### Cell survival and mortality

In an in-vitro experiment by Ortona et al. [56], cardiac cells (AC16 cell line) were treated with 1 μM doxorubicin for 72 h, and it was observed that cardiac cell survival following the chemotherapeutic drug administration was significantly lower than that of the untreated cells. In contrast, the findings showed that pretreated with 100 μM silibinin for 48 h could protect the cardiac cells against doxorubicin-induced reduction in cell survival [56].

Two in vivo experiments revealed that the mortality rate in the doxorubicin-treated rats/mice was higher than that in the control groups [57, 58]. However, the use of silymarin remarkably reduced the doxorubicin-induced mortality rate [57]. Patel et al. reported that a single dose of 60 mg/kg doxorubicin caused 55% death in mice, while the silymarin co-administration (16 mg/kg/day, for 14 days) decreased lethality induced by doxorubicin from 55 to 9% [57].

#### Body weight and heart weight changes

The results of in-vivo studies showed that the body weight and heart weight of mice/rats treated with doxorubicin were lower than those of the control groups [57–60]. A significant accumulation of ascites, pericardial, pleural, and peritoneal fluids in the animals treated with doxorubicin in comparison with the untreated group was also found [58]. Other findings indicated that the silymarin co-administration could restore the body weight and heart weight of doxorubicin-treated mice/rats [57–60].

#### Electrocardiography (ECG) changes

In an in-vivo experiment, it was observed that doxorubicin-treated rats had several ECG changes consisting of bradycardia and prolongation of QT and QRS interval. However, these ECG abnormalities were obviously improved in the animals receiving silymarin plus doxorubicin [58].

In a clinical study, the echocardiographic examinations of children with acute lymphoblastic leukemia were obtained before and after doxorubicin treatment alone and in combination with silymarin. According to the findings, a significant reduction in ejection fraction, tissue Doppler peak mitral annulus systolic velocity, and fractional shortening of the cancer patients were observed following doxorubicin administration. Moreover, the cancer patients receiving silymarin plus doxorubicin showed a significant increase in these parameters evaluating systolic function compared to the doxorubicin group alone [61].

#### Biochemical changes

The findings obtained from some studies showed that the doxorubicin administration could induce biochemical changes in the cardiac cells/tissue, as listed in Table 1. Briefly, it was shown that the lactate dehydrogenase (LDH), creatine kinase, aspartate aminotransferase (AST), creatine phosphokinase (CPK), troponin-I, creatine kinase-myocardial band (CK-MB), reactive oxygen species (ROS), malondialdehyde (MDA), thiobarbituric acid reactive substances (TBARS), nitrite, nitric oxide, hydrogen peroxide (H_2_O_2_), inducible nitric oxide synthase (iNOS), caspase-3, tumor necrosis factor-alpha (TNF-α), nuclear factor erythroid 2-related factor 2 (Nrf2), vascular endothelial growth factor A (VEGF-A), plasma cholesterol, total lipids, total cholesterol, triglycerides, low-density lipoprotein-cholesterol (LDL-c), triglyceride/high-density lipoprotein (TG/HDL), LDL/HDL, and C-reactive protein levels significantly elevated in the doxorubicin-treated groups than the untreated/control groups [56–66]. Additionally, the glutathione peroxidase (GPx), glutathione (GSH), superoxide dismutase (SOD), catalase, peroxidase, glutathione reductase, gamma-glutamyl transferase (γ-GT), glutathione-S-transferase (GST), HDL-c, and interleukin-10 (IL-10) levels significantly decreased following the doxorubicin treatment than the untreated/control groups [60, 64–66].

Other results also indicated that, for most of the cases, the silymarin/silibinin co-administration could alleviate the doxorubicin-induced biochemical alterations in the cardiac cells/tissue [34, 56–67].

#### Histological and ultrastructural changes

The histopathological and ultrastructural examinations of heart sections of the doxorubicin-treated mice/rats indicated the following tissue changes: necrotic muscle fibers, hypertrophy of muscle fibers, wide spaces between muscle fibers, cytoplasmic vacuole formation, highly eosinophilic cytoplasm, disturbance in cardiac trabeculae, interstitial edema, mild hyperemia, vascular congestion, myofibrillar disorganizations, infiltration of inflammatory cells, increase in number of focal necrosis and fibrosis (%), disintegration and dilatation of sarcoplasmic reticulum, vesiculated rough endoplasmic reticulum, eosinophilic degeneration, distorted blood capillaries, severe hemorrhage, retrogressive lacerations in muscle fibers, degenerated cardiac myocytes with small deeply stained pyknotic nuclei and vacuolated cytoplasm, thickening of coronary artery wall, degenerated cardiac myocytes with irregular corrugated thick basement membrane, cardiac myocytes with small shrunken fragmented nucleus, cardiac myocytes with wide intercellular space containing many fibroblasts and collage fibers, and so on [58–60, 62–66].

It was also observed that the silymarin/silibinin co-administration could mitigate the doxorubicin-induced histological/ultrastructural changes in the cardiac tissue [58–60, 62–66].

## Discussion

**Fig. 2:**
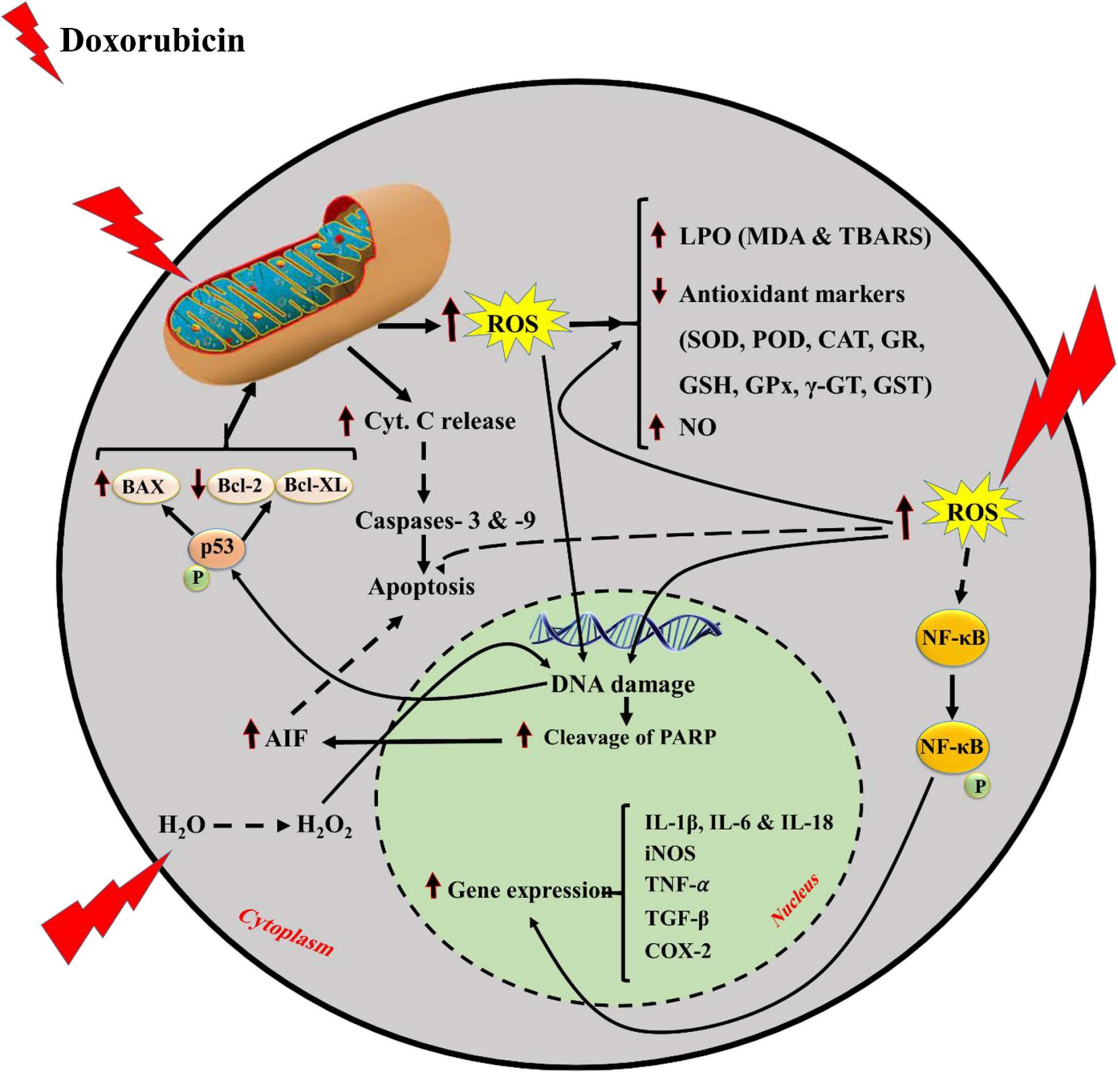
The molecular mechanisms of cardiac damage induced by doxorubicin. The doxorubicin administration leads to induction of oxidative damage, mitochondria damage, apoptosis, inflammation, and other mechanisms in the cardiac cell. In contrast, the silymarin/silibinin co-administration, through an opposite pattern, alleviates the doxorubicin-induced cardiac cell injury. ↓decreased by doxorubicin; ↑increased by doxorubicin; MDA, malondialdehyde; TBARS, thiobarbituric acid reactive substances; SOD, superoxide dismutase; POD, peroxidase; CAT, catalase; GR, glutathione reductase; GSH, glutathione; GPx, glutathione peroxidase; γ-GT, gamma-glutamyl transferase; GST, glutathione-S-transferase; NO, nitric oxide; ROS, reactive oxygen species; NF-κB, nuclear factor kappa B; IL, interleukin; iNOS, inducible nitric oxide synthase; TNF-α, tumor necrosis factor-alpha; TGF-β, transforming growth factor-beta; COX-2, cyclooxygenase-2; BAX, Bcl-2-associated X protein; AIF, apoptosis-inducing factor; PARP, poly (ADP-ribose) polymerase

In the current study, the effects of doxorubicin therapy alone and in combination with silymarin/silibinin on normal cardiac cells/tissue are reviewed and the findings extracted from the eligible studies are summarily presented in Table 1. Furthermore, some of the important effects of doxorubicin alone and silymarin/silibinin plus doxorubicin on the cardiac cell are shown in Fig. 2.

The cardiac insult, myocardial infarction, and tissue ischemia can be detected by estimation of recognized cardiac marker enzymes, including cholesterol, creatine kinase, CPK, CK-MB, LDH, and AST present in the serum [68, 69]; hence, the activity assessment of these enzymes is important for prediction of cardiac damage. Some studies have reported that the doxorubicin administration significantly elevated the serum activities of these heart damage-associated enzymes, which were released from the damaged cardiac cells [57–60, 62, 64, 65]. It was reported that the increased serum level of troponin I shortly following chemotherapy can be considered as a powerful predictor for ventricular dysfunction and poor cardiac outcome [61, 70, 71]. Nevertheless, the co-administration of silymarin/silibinin could reduce the elevated serum levels of heart damage-associated enzymes (cholesterol, creatine kinase, CPK, CK-MB, LDH, and AST) and cardiac troponin I in the doxorubicin-treated groups [57–62, 64, 65].

It has been also shown that the doxorubicin administration might affect hematological parameters such as induction of anemia, reduction of platelet numbers, increase of lymphocyte numbers, decrease of hemoglobin concentration, etc. [60, 72, 73]. In a study by Afsar et al. it was reported that the silymarin co-administration resulted in a significant improvement in the hematological parameters of doxorubicin-treated rats [60].

Cardiac adverse effects are closely related to oxidative stress caused by excessive free radicals (such as ROS), lipid peroxidation (LPO), and antioxidant depletion [74]. The semiquinone form of doxorubicin is able to interact with molecular oxygen for ROS generation in cardiac cells [59]. The doxorubicin-generated ROS attack the cell macromolecules (such as DNA, RNA, and lysosome), leading to the malfunction of the heart tissue [75–79]. Moreover, the doxorubicin administration causes LPO, an interaction between doxorubicin-generated free radicals and unsaturated fatty acids normally in membrane lipids [57, 80, 81]. The TBARS and MDA levels have been reported to be a credible marker of LPO; in this regard, some studies have reported that the doxorubicin administration increased the TBARS and MDA levels of cardiac cells/tissue [60, 62, 66, 82]. Furthermore, the antioxidant endogenous system (including SOD, peroxidase, catalase, glutathione reductase, GSH, GPx, γ-GT, GST) provides defense against the oxidative damage through neutralizing additional free radicals [60, 83–85]; nevertheless, it was revealed that these endogenous antioxidant levels decreased in the doxorubicin-treated cardiac cells/tissue [58, 60, 64, 66, 82, 86–93]. The H_2_O_2_ level also increased in rats treated with doxorubicin [60]. Additionally, there is normally a low amount of nitric oxide in the cardiac cells [23]. It was reported that the nitric oxide level of cardiac cells increased following doxorubicin treatment and this free radical has notable roles in cellular signaling during pathological processes [94, 95]. The superoxide anion (O_2_^−^) produced from an oxygen molecule following doxorubicin treatment highly interacts with nitric oxide, which can produce peroxynitrite (ONOO^−^) [96]. Moreover, the ONOO^−^ can turn to other reactive nitrogen species (RNS), including NO_2_^−^, NO_3_^−^, OH^−^, and CO_3_^−^ [23]. The mitochondria injury following doxorubicin via mitochondria ROS production has been reported previously [56, 97]. Doxorubicin has also a high binding affinity to cardiolipin in the inner mitochondria membrane, directly leading to the electron transport chain disturbance, which causes excessive ROS and RNS [98–100]. It has been shown that silymarin through its antioxidant effects can inhibit oxidative stress by scavenging free radicals and increasing cellular antioxidant defense mechanisms [101–105]. Moreover, silymarin is able to decrease LPO and its anti-lipoperoxidation activity can be due to the presence of taxifolin and the ability of its polyphenols to bind transition metals and quench ROS [34]. Furthermore, the increased levels of oxidative stress markers (MDA, TBARS, nitric oxide, and H_2_O_2_) and the reduced levels of antioxidant markers (SOD, peroxidase, catalase, glutathione reductase, GSH, GPx, γ-GT, GST) in the doxorubicin-exposed cardiac cells was reversed by the silymarin/silibinin co-administration [34, 60, 62–64, 66]. It was also shown that the co-treatment of silibinin reduced mitochondrial ROS generation, mitochondria membrane depolarization, and cytoskeleton changes associated with doxorubicin in cardiomyocytes [56].

Doxorubicin also stimulates apoptosis via both intrinsic and extrinsic pathways [106, 107]. This chemotherapeutic agent leads to excess oxidative stress and mitochondrial damage, triggering apoptotic cell death [108–111]. Some important mediators involved in the apoptotic process are p53, B-cell lymphoma-extra large (Bcl-xL), Bcl-2, BAX, cleaved poly (ADP-ribose) polymerase (PARP), caspase enzymes, and so on [23, 112–117]. Some studies have reported that doxorubicin chemotherapy upregulates BAX, cleaved caspase-3, cleaved caspase-9, and p53 expressions and downregulates Bcl-2 and Bcl-xL expressions in the cardiac cells [75–77, 118–124]. These findings indicate that the cells are moving toward apoptotic cell death. It has been also reported that doxorubicin via activation of c-Jun N-terminal kinase (JNK) and p38 mitogen-activated protein kinases (MAPKs) pathways can trigger cardiac apoptosis [125]. The anti-apoptotic effects of silymarin/silibinin have been reported in previous studies. In this regard, it was shown that silymarin is able to prevent the release of cytochrome c, thereby inhibiting the activation of caspases [126, 127]. Additionally, the silymarin/silibinin treatment increased the Bcl-2 and Bcl-xL levels and decreased the BAX, p53, JNK and p38 MAPKs, PARP, and caspase-3 levels in the cells [29, 56, 57, 64, 105, 128–131].

The cancer chemotherapy may trigger an inflammatory process [132], leading to the incidence of various adverse effects following this therapeutic modality [133]. Some studies have reported that the cancer chemotherapy with doxorubicin can cause cardiac inflammation [89, 90, 134, 135]. The inflammatory process is positively correlated with oxidative stress in cardiotoxicity [74]. It has been reported that doxorubicin-induced oxidative stress can activate lysosomal enzymes, leading to the promotion of cardiac inflammation [23]. According to the findings obtained from some studies, it was indicated that doxorubicin treatment led to an increase in the production of pro-inflammatory mediators (iNOS, COX-2, TGF-β, IL-1β, IL-6, IL-18, NF-κB, and TNF-α) and a reduction in IL-10 level (an anti-inflammatory cytokine) of cardiac cells [64, 66, 75, 82, 120, 122, 135]. Previous studies have reported that silymarin/silibinin can be a promising anti‐inflammatory agent. It was shown that the use of silymarin/silibinin could reduce the inflammation via decreased levels of iNOS, COX-2, TGF-β, IL-1β, IL-6, IL-18, and TNF-α along with an increased level of IL-10 in different cells/tissues [64, 66, 128, 136–141]. Moreover, the anti-inflammatory effects of silymarin can mainly be because of inhibiting the NF-κB nuclear translocation/activation, resulting in preventing the aggregation of inflammatory cells as well as decreasing the expression of inflammatory cytokines and other certain inflammatory mediators [105, 128, 131, 142–144]. In addition, the histological findings represented in this systematic review exhibited that the doxorubicin-induced cardiac inflammation is mitigated by the silymarin/silibinin co-administration [58, 60, 62–65].

## Perspective of future research and limitations

Although the doxorubicin chemotherapy is commonly applied for treating the cancer patients, its cardiotoxic adverse effects limit the clinical application of this chemotherapeutic agent. According to the data presented in this systematic review, it was shown that silymarin/silibinin can be an effective cardioprotective agent against the doxorubicin-induced cardiotoxicity. This herbal agent exerts the cardioprotective activities via the antioxidant, anti-apoptotic, anti-inflammatory effects, and other mechanisms. In addition to its chemo-protective effects, silymarin/silibinin can be used as a chemosensitizing agent on cancerous cells, mitigating the chemotherapy-induced adverse effects via reduction of the chemotherapy dose in the cancer patients.

Despite its remarkable beneficial effects, it has been reported that silymarin has very low water solubility and poor oral absorption. A number of researchers have overcome these biopharmaceutical drawbacks by using various structural modification strategies [145–147] and have introduced novel derivatives and analogues for silymarin [148–156]. Furthermore, the therapeutic/protective efficacy of novel derivatives/analogues has been investigated on tumor/normal cells [148, 150, 157, 158]. Other researchers have reported that the loading of silymarin into a delivery system improves its bioavailability; hence, they developed various formulation-based approaches such as solid lipid nanoparticles, mesoporous silica nanoparticles, biodegradable polymeric micelles, nanoemulsions, amorphous solid dispersions, nanosuspensions, and liposomes [159–165]. Some studies have assessed the therapeutic/protective effects of silymarin delivery systems on tumor/normal cells [166–170]. In view of the above, evaluating the potential cardioprotective potentials of the analogues/derivatives and the delivery systems of silymarin/silibinin against cardiotoxicity induced by chemotherapy drugs (especially doxorubicin) is suggested.

Since the data represented in this study are mostly based on in vitro and in vivo experiments, suggesting the use of silymarin/silibinin (as a potential cardioprotective agent) in the cancer patients for alleviating the cardiac adverse effects induced by doxorubicin or other chemotherapy drugs requires further clinical studies. Moreover, another point that should be evaluated with more extensive studies on the current topic is to provide more details on the type of cancer, the dose and frequency of administration of the drugs.

## Conclusion

The findings reveal that the doxorubicin chemotherapy could induce echocardiographic, biochemical, and histological alterations in the cardiac cells/tissue which caused cardiotoxicity. Other results showed that the silymarin/silibinin co-administration could alleviate the doxorubicin-mediated cardiac adverse effects. Mechanically, the silymarin/silibinin exerts its cardioprotective effects via the antioxidant, anti-apoptotic, anti-inflammatory effects, and other mechanisms.

## Data Availability

The datasets used and/or analyzed during the current study are available from the corresponding author on reasonable request.
